# Focal spot motion in digital breast tomosynthesis and its effect on spatial resolution

**DOI:** 10.1002/acm2.70443

**Published:** 2026-01-09

**Authors:** Colin J. Schaeffer, Katie W. Hulme, Ashley E. Rubinstein

**Affiliations:** ^1^ Department of Diagnostic Radiology Oregon Health & Science University Portland Oregon USA; ^2^ Department of Radiology Cleveland Clinic Cleveland Ohio USA; ^3^ Department of Radiology Henry Ford Health Detroit Michigan USA

**Keywords:** focal spot blur, mammography, tomosynthesis

## Abstract

**Background:**

Digital breast tomosynthesis (DBT) has become standard practice; however, the acquisition method of DBT between vendors is far from standardized. Currently, there are three commercially available DBT tube motion techniques: (1) continuous motion, (2) step‐and‐shoot, and (3) continuous motion with flying focal spot. Each of these methods represents a trade‐off between total acquisition time and focal spot blur.

**Purpose:**

The aim of the study was to characterize the increase in effective focal spot size in DBT relative to standard 2D projections and assess the influence of this increase on spatial resolution using the modulation transfer function (MTF).

**Methods:**

Focal spot size was measured for both a 2D acquisition and the 0° DBT projection using a 10 µm slit phantom. Imaging techniques were set to those used for a 2, 4, and 8 cm thick breast of 50/50 adipose/fat composition. MTF curves were measured using a copper edge phantom both at the breast support plane and 4 cm above the breast support.

**Results:**

The effective focal spot size increase from 2D to DBT increased with breast thickness for all systems. The continuous motion systems showed the greatest increase in effective focal spot size with percent increases of 101% to 462% depending on unit and breast thickness. The flying focal spot system showed the smallest increase in effective focal spot size in DBT acquisitions, being 3%, 21%, and 25% for a 2, 4, and 8 cm thick breast, respectively. The step‐and‐shoot and flying focal spot systems showed no degradation in MTF curves due to increasing effective focal spot size in DBT acquisitions, while the continuous motion systems showed a reduction in the frequency at which the MTF curve reached 50% of 26%–45%.

**Conclusion:**

Both step‐and‐shoot and flying focal spot systems minimized effective focal spot size increase in DBT acquisitions compared to continuous tube motion systems.

## 1 INTRODUCTION

Mammography is a powerful screening and diagnostic tool that has contributed significantly to the reduction in breast cancer mortality.^1^ However, despite its effectiveness, 2D mammography is limited by superposition of anatomy that reduces sensitivity for women with dense breasts.^2^

Digital breast tomosynthesis (DBT) was introduced in 2011 to overcome this limitation. DBT creates a pseudo‐3D stack of images reconstructed from multiple projections taken at different angles respective to the breast. Since its introduction, DBT has become a well‐established practice, with DBT often accompanying 2D screening exams or even supplanting 2D in favor of DBT‐based synthetic 2D images.^3^ While DBT is now standard practice, its acquisition is far from standardized with vendors opting for different angular ranges, number of projections, and tube motion techniques. These differences impact image quality in different ways. Of note for this study, is the impact that tube motion has on spatial resolution of the DBT projections. Currently, there are three approaches to tube motion in DBT: (1) continuous, (2) step‐and‐shoot, and (3) continuous motion with flying focal spot (henceforth simply referred to as flying focal spot). Each of these techniques has differing effects on the effective focal spot size of the projections and, in turn, differing effects on spatial resolution.

With more sites switching to DBT‐only screening, it is important to understand the degradation effects of these different implementations. The aim of this study was to examine the effective focal spot size for all three currently commercially available DBT tube motion techniques, as well as to assess the effect that effective focal spot size has on spatial resolution in the projected images.

## 2 METHODS

To examine focal spot motion in DBT, we took a two‐tiered approach. First, we looked at the effective focal spot size for all three DBT acquisition types and compared them to their respective 2D acquisition focal spot size. Next, we measured MTF curves for all DBT acquisition types for 2D and DBT projections both on the breast support plate and 4 cm above the breast support plate. While both MTF curves are measures of system resolution at the plane of measurement, comparing the MTF curves measured on the breast support to those measured 4 cm above the breast support isolated the effect of resolution degradation due to geometric influences, primarily focal spot blur. All images, 2D and DBT projections, used for analysis were the for‐processing images.

The magnitude of focal spot blur in DBT is highly shift variant and depends on the spatial location of measurement. In this study, all measurements of focal spot size were taken on the central ray of the x‐ray field to give a consistent measuring point for comparing across systems. For a complete review on the spatial dependence of focal spot blur, we refer the reader to a study by Zheng et al.^4^ Though the spatial variance of the effective focal spot was not investigated, the mAs dependence was evaluated by measuring focal spot size at techniques representative of those used to image a 2, 4, and 8 cm thick breast of 50/50 adipose/glandular composition.

### 2.1 Mammography units

The mammography units included in this study are listed in Table 1. We included units from three major mammography vendors: Hologic, GE, and Siemens. These units also encompass the three current DBT tube motion approaches: continuous motion, step‐and‐shoot, and flying focal spot. Tables 2 and 3 show the imaging techniques used in this study.

**TABLE 1:** Mammography units included in study.

Unit	DBT acquisition	Pixel size (µm)	Angular range	# of projections	DBT scan time (s)
**Hologic**					
3Dimensions	Continuous	70	15°	15	3.7
Selenia dimensions	Continuous	70 (140^*^)	15°	15	3.7
**GE**					
Senographe pristina	Step‐and‐shoot	100	25°	9	10
Senographe essential	Step‐and‐shoot	100	25°	9	10
**Siemens**					
Mammomat B.brilliant	Flying focal spot	85	50°	25	5
Mammomat revelation	Continuous	85	50°	25	25

* System bins detector elements in DBT acquisition.

**TABLE 2:** Imaging techniques used to measure focal spot size in 2D acquisitions.

Unit	2 cm			4 cm			8 cm		
Parameter	T/F^a^	kVp	mAs	T/F^a^	kVp	mAs	T/F^a^	kVp	mAs
**Hologic**									
3Dimensions	W/Rh	25	60	W/Rh	28	120	W/Ag	32	360
Selenia dimensions	W/Rh	25	60	W/Rh	28	120	W/Ag	32	360
**GE**									
Senographe pristina	Mo/Mo	26	16	Rh/Ag	34	32	Rh/Ag	34	110
Senographe essential^b^	–	–	–	Mo/Rh	27	125	–	–	–
**Siemens**									
Mammomat B.brilliant	W/Al	24	45	W/Al	26	71	W/Al	28	220
Mammomat revelation	W/Rh	26	36	W/Rh	27	71	W/Rh	31	360

^a^ T/F = target/filter.

^b^ System focal spot was not measured.

**TABLE 3:** Imaging techniques used to measure focal spot size in DBT acquisitions.

Unit	2 cm				4 cm				8 cm			
Parameter	T/F^a^	kVp	mA	PW^c^	T/F^a^	kVp	mA	PW^c^	T/F^a^	kVp	mA	PW^c^
**Hologic**												
3Dimensions	W/Al	26	140	16.7	W/Al	29	165	22.4	W/Al	38	180	37.1
Selenia dimensions	W/Al	26	140	14.4	W/Al	29	165	20.3	W/Al	38	180	37.1
**GE**											
Senographe pristina	Mo/Mo	26	40	50	Rh/Ag	34	40	69	Rh/Ag	34	54	183
Senographe essential^b^	–	–	–	–	Rh/Rh	29	63	79	–	–	–	–
**Siemens**											
Mammomat B.brilliant	W/Al	25	79	22.8	W/Al	26	172	23	W/Al	31	156	32
Mammomat revelation	W/Rh	26	27	94	W/Rh	28	58	88	W/Rh	31	161	112

^a^ T/F = target/filter.

^b^ System focal spot was not measured.

^c^ PW = pulse width in ms per DBT projection.

### 2.2 Focal spot measurements

**FIGURE 1:**
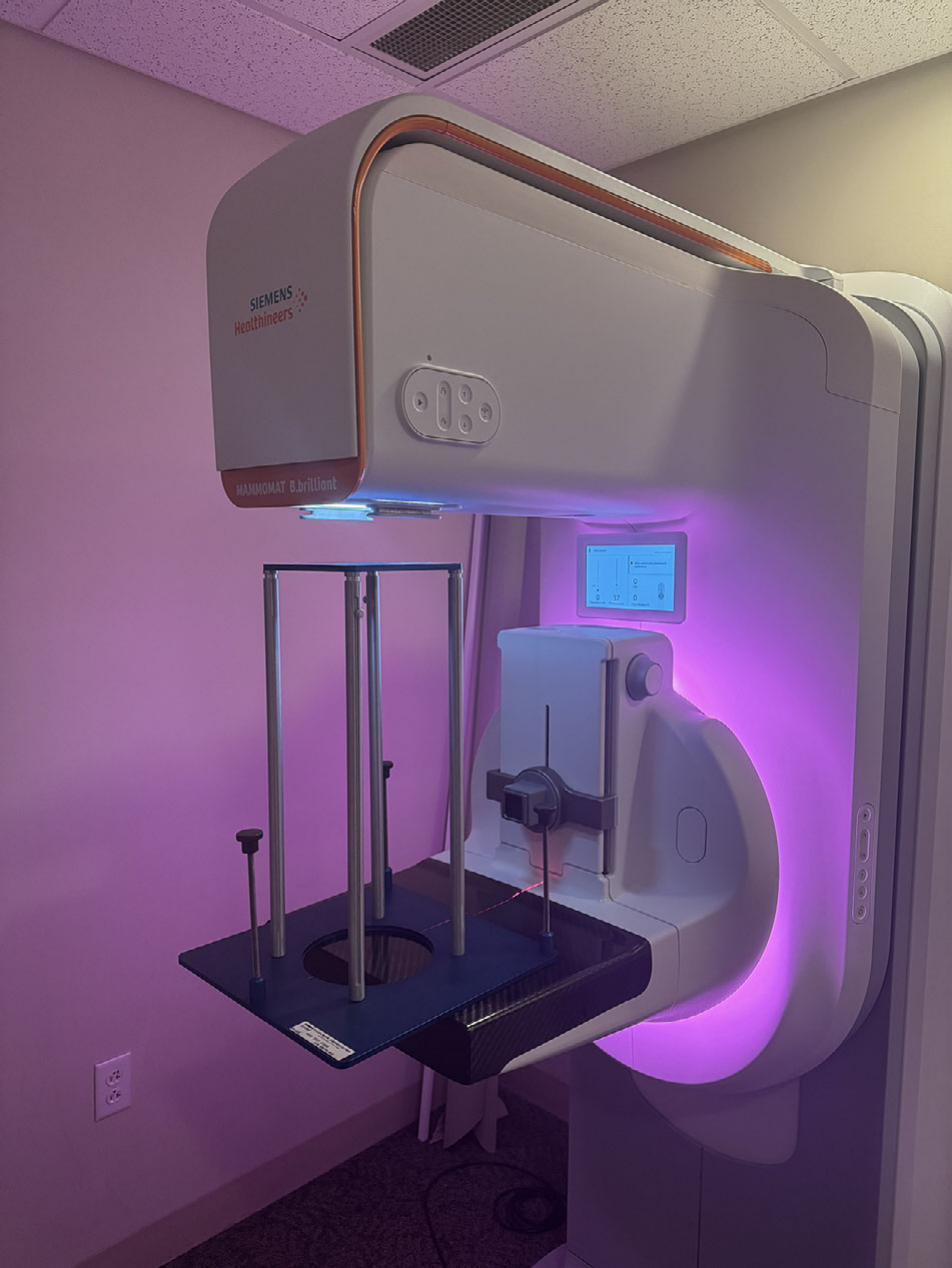
Image showing setup of focal spot test stand on a Siemens B.brilliant mammography system.

**FIGURE 2:**
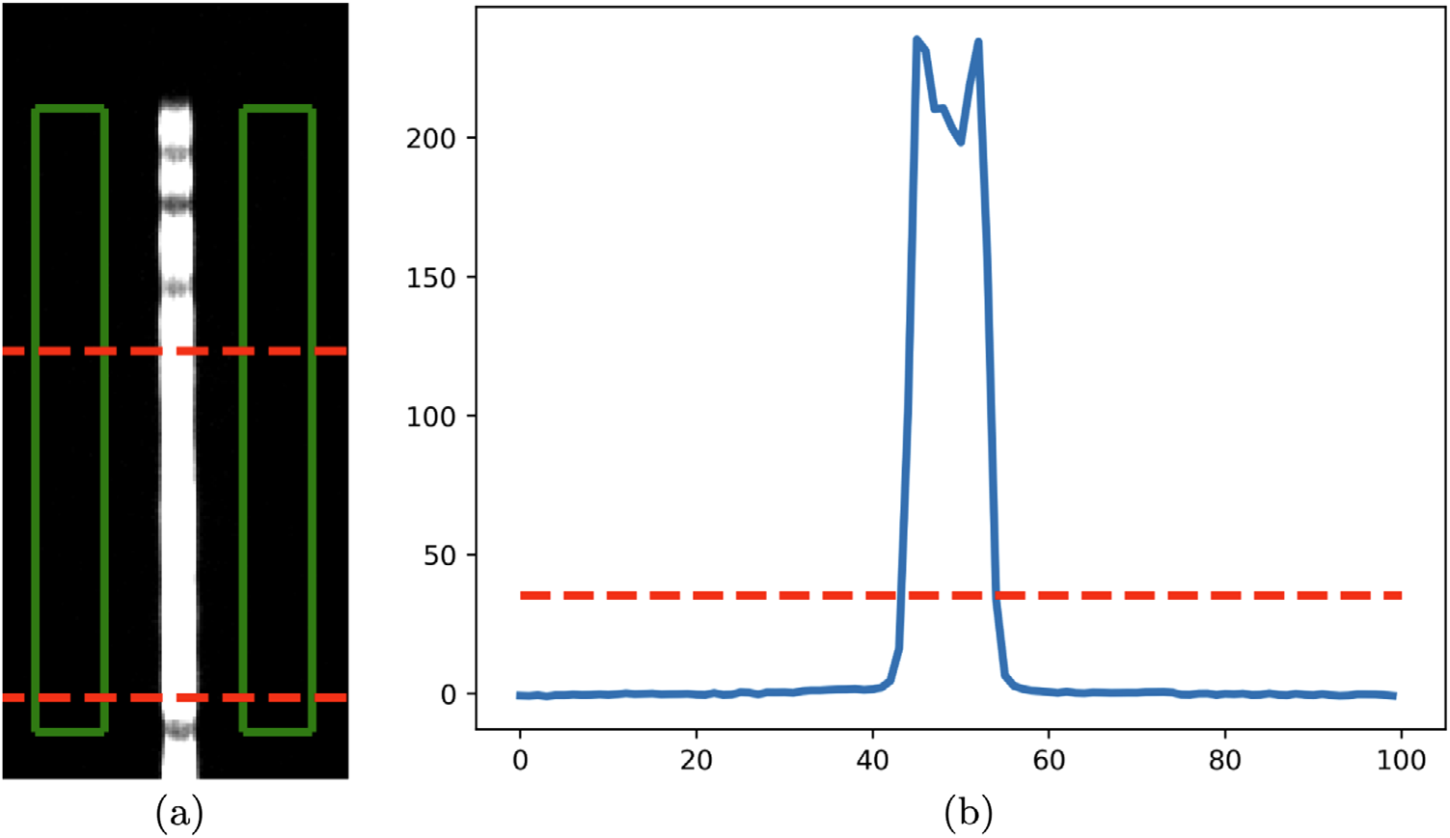
Workflow to extract focal spot width from image of slit phantom. (a) Cropped, zoomed‐in projection of slit phantom with green boxes to show area used for background subtraction and red lines to show area over which line profiles were averaged, (b) averaged, background‐subtracted line profile with red line marking width at 15% max.

Focal spot size was measured using a 10 µm slit phantom and focal spot test stand, shown in Figure 1 (Fluke Biomedical product 07–622 and 07–624).^5^ Focal spot size for 2D acquisitions were measured in a 2D acquisition mode while the effective focal spot size in DBT acquisitions were measured using the 0° projection. Images of the slit phantom were acquired with techniques consistent with those used to image a 2, 4, and 8 cm thick breast of 50/50 glandular/adipose composition as determined by the AEC test at the annual physics evaluations of each system using the methodology of the ACR QC manual.^6^ Three measurements were taken for each set of techniques on each system with complete tear down and reassembly of the alignment stand in between measurements to include variance influenced by apparatus setup. The process for calculating focal spot size from projections of the slit phantom is described below:

1. Align test stand with central ray of x‐ray field using the alignment phantom.
2. Calculate magnification using the magnification phantom.
3. Insert 10 µm slit phantom and take projection to verify angle of slit is less than 1°.
4. Average multiple line profiles across slit projection and subtract background (shown in Figure 2a).
5. Measure line spread width at 15% maximum, as recommended by the IEC (shown in Figure 2b).^7^
6. Adjust for magnification by Equation  (1).
(1) $$FSS\left(mm\right)=\frac{FW15M-0.01M}{M-1}$$

where *FSS* is the focal spot size, *FW15M* is the full‐width at 15% maximum, and *M* is the magnification factor measured in step 2. Details on the alignment phantom, magnification phantom, and process can be found on the Fluke Biomedical product manual.^5^

It is important to note that we did not meet the criteria stated in IEC 60336:2020 on focal spot measurements; namely the requirement of a 30‐pixel minimum sampling of the line spread of the slit phantom.^7^ Because of this and the different sampling rates between models due to varying pixel pitch, it is not appropriate to compare our measured results against the stated focal spot size provided by the manufacturer or to report absolute size of the measured effective focal spot for inter‐unit comparison. However, since measurement methodology remained consistent between 2D and DBT measurements on each specific unit, it is still valid to compare these results as a measure of relative increase in effective focal spot size in DBT compared to 2D acquisitions. Therefore, only percent increase in effective focal spot size, from 2D to DBT projections for the same breast thickness, is reported in this study. Focal spot size was only measured in the direction of DBT motion, that is parallel with the chest wall.

Focal spot measurements were not taken on the GE Senographe Essential. The alignment/magnification stand did not fit under the c‐arm gantry, and therefore sufficient magnification of the slit phantom could not be achieved.

### 2.3 MTF measurements

MTF curves were measured and calculated from a 1 mm thick straight, polished copper edge following the methodology of Samei et al.^8^ To isolate the effect of focal spot blur, MTF curves were calculated both with the copper edge on the breast support plate and 4 cm above the breast support plate. MTF curves, as well as the frequency at which the curve drops to 50%, were reported. MTF measurements were only performed for techniques used to image a 4 cm thick breast of 50/50 adipose/glandular composition and all spatial frequencies were calculated at the plane of the detector. The ROI size used for calculation of the MTF varied slightly between units but was as such to cover a 2 cm × 4 cm area at the detector plane with the long dimension perpendicular to the chest wall. Each ROI started 2 cm from the chest wall and extended away from the chest wall.

## 3 RESULTS

### 3.1 Focal spot measurements

The relative increase in focal spot size from 2D to DBT acquisitions for each unit are shown in Table 4. The percent increase varied from 1% to 462% and trended upwards with increasing pulse width of the DBT projection. Though always monotonic, the relationship between pulse width of the DBT projection and effective focal spot size increase varied between units.

**TABLE 4:** Percent focal spot size increase from 2D to DBT projection. Parenthesis show standard deviation of three measurements.

Unit	2 cm	4 cm	8 cm
**Hologic**			
3Dimensions	101% (6%)	182% (24%)	312% (3%)
Selenia dimensions	105% (4%)	189% (19%)	332% (30%)
**GE**			
Senographe pristina	1% (14%)	32% (9%)	60% (3%)
Senographe essential	–	–	–
**Siemens**			
Mammomat B.brilliant	3% (2%)	21% (5%)	25% (2%)
Mammomat revelation	254% (6%)	320% (10%)	462% (35%)

The DBT acquisition type that produced the largest percent increase in effective focal spot size was the continuous tube motion technique. This was followed by the step‐and‐shoot and flying focal spot techniques, which both produced similar increases in effective focal spot sizes. The flying focal spot technique had a smaller increase at the imaging techniques used to image a 4 and 8 cm thick breast.

### 3.2 MTF measurements

**FIGURE 3:**
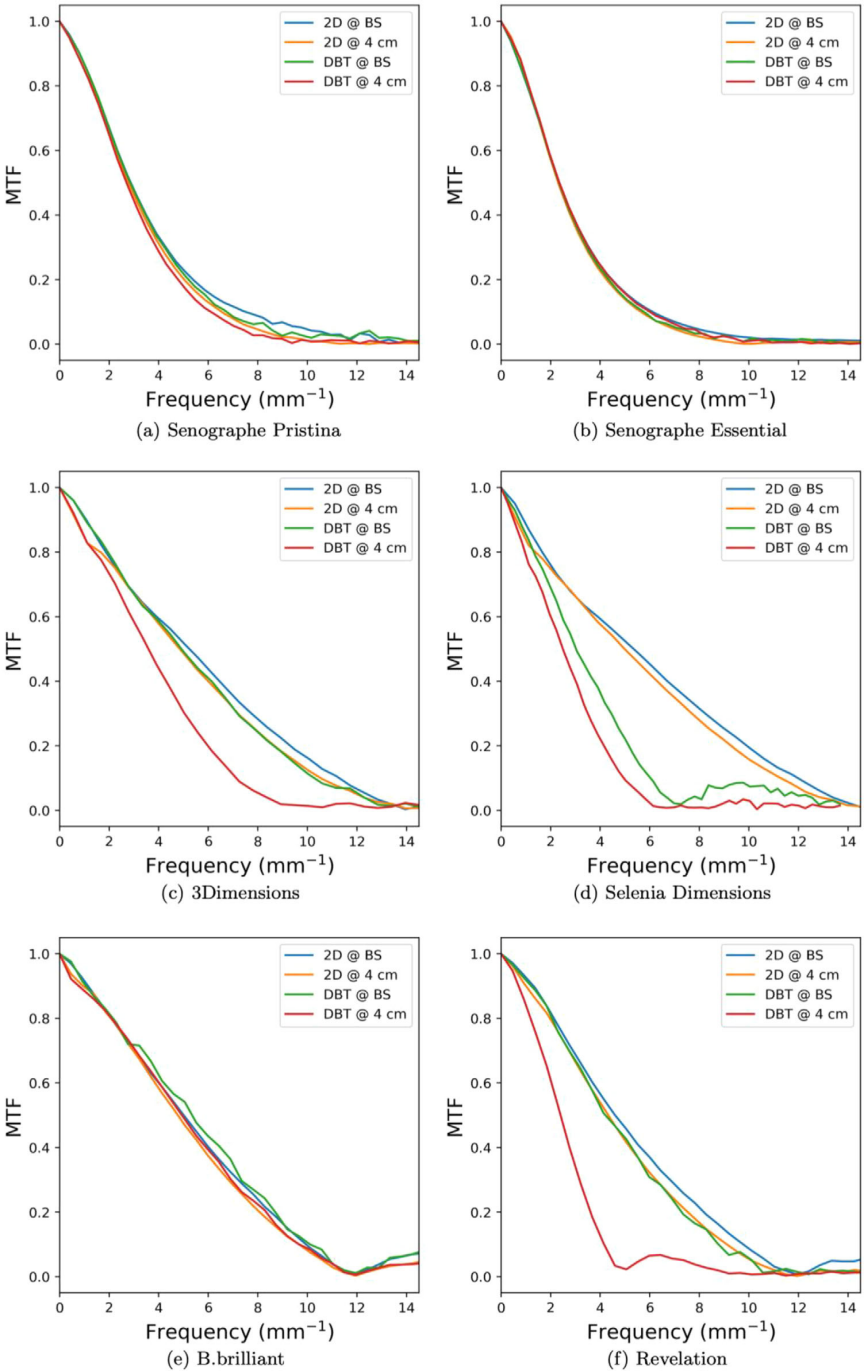
MTF curves of all mammography units included in this study.

Measured MTF curves and the frequency values at which these curves reached 50% are shown in Figure 3 and Table 5, respectively. Also shown in Table 5 is the percent reduction in MTF50 frequency, where a positive percent reduction indicates a reduced spatial frequency at which the curve hits 50%. Several systems showed a negative percent reduction in MTF50, which was likely due to noise in the measurement. The 2D MTF curves showed slight resolution degradation due to geometric blur when comparing the curves at the plane of the breast support to those 4 cm above the breast support. Spatial resolution degradation between 2D acquisitions and the central projection in a DBT acquisition varied between units. The continuous motion systems showed the greatest amount of spatial resolution loss due to focal spot size increase with the 3Dimensions and Revelation systems showing a 26% and 45% decrease in the MTF50 frequency, respectively. Conversely, the step‐and‐shoot and flying focal spot systems showed little spatial resolution loss in DBT compared to 2D.

**TABLE 5:** Frequency values (mm^−1^) at which the MTF drops to 50% for MTF curves measured in the planes directly above the breast support plate and 4.0 cm above the breast support plate for both 2D and DBT projections. Also shown is the percent reduction of the 50% MTF frequency of DBT projection compared to the respective 2D projection.

	Breast support plane			4 cm above breast support		
Unit	2D	DBT	% Reduction	2D	DBT	% Reduction
**Hologic**						
3Dimensions	5.24	4.92	6%	4.85	3.59	26%
Selenia dimensions	5.35	3.03	43%	4.98	2.49	50%
**GE**						
Senographe pristina	2.87	2.86	1%	2.79	2.70	3%
Senographe essential	2.34	2.34	1%	2.30	2.35	−2%
**Siemens**						
Mammomat B.brilliant	5.01	5.35	−7%	4.77	4.93	−3%
Mammomat revelation	4.60	4.21	8%	4.33	2.40	45%

## 4 DISCUSSION

In this study we measured and compared the effective focal spot size increase, and its effect on spatial resolution, due to different DBT acquisition techniques. We found that for clinical techniques, both the step‐and‐shoot and flying focal spot techniques produced effective focal spot sizes that were 1%–60% larger than the effective focal spot size used for 2D acquisitions for the same breast thickness. This was significantly less than the increase observed for continuous tube motion systems. For these systems, the effective focal spot size increase ranged from 101% to 462%. The MTF curves verified that the marginal increase in focal spot size for the step‐and‐shoot and flying focal spot systems did not significantly affect spatial resolution, while the continuous tube motion systems showed noticeable degradation of the system MTF curves.

Despite the increase in focal spot size in continuous tube motion systems, the 3Dimensions still showed higher spatial resolution than both step‐and‐shoot systems as well as other continuous tube motion systems due to its direct conversion a‐Se detector with a 70 µm pixel pitch. The flying focal spot B.brilliant system with its 85 µm pixel pitch and direct conversion a‐Se detector gave the best DBT projection MTF curves among all systems included in this study. These results are consistent with a study by Houbrechts et al. who first published a characterization of the flying focal spot system.^9^ The GE system with its 100 µm pixel pitch gave the lowest 2D MTFs and DBT MTFs that were only higher than the Siemens Revelation and the binning acquisition of the Selenia Dimensions. These results highlight that resolution is a combination of focal spot blur and detector blur and that if focal spot blur is too high, the system can give worse spatial resolution than a system with a larger pixel pitch (i.e., GE Pristina DBT vs. Siemens Revelation DBT MTF at 4 cm above breast support). Regardless of tube motion technique, this study found that effective focal spot size increase from 2D to DBT increased with breast thickness. We hypothesize that this was largely due to the increasing pulse width of the DBT projections with increasing breast thickness. Increasing pulse width increased focal spot blur for both continuous and flying focal spot systems and gave more time for vibrations to affect the focal spot size for step‐and‐shoot systems.

The impact of our findings on cancer detection is unclear, and it is possible that any resolution degradation due to effective focal spot size increases may be masked by DBT reconstruction methods. Though we can make no definitive statements, the reduction of spatial resolution in DBT projections of continuous tube motion systems compared to their respective 2D projections may imply reduced conspicuity of fine structures. This may have an increased impact on clinical care with the increased use of synthesized 2D mammograms generated from DBT projections.

This study had multiple limitations. First, we did not report absolute focal spot size measurements. Vendors state focal spot size in accordance with IEC 60336:2020. Our imaging techniques and digital sampling rate did not strictly adhere to that document, so we refrained from reporting absolute focal spot size measurements. Furthermore, since the digital sampling rate (i.e. pixel pitch) varied between units, we felt absolute focal spot size was not a valid comparison between units. Instead, since measurement methodology was consistent for measurements made on a particular system, we reported percent increase in effective focal spot size as our comparison metric between units.

Our next limitation was that we only took measurements on one unit per model. Though we believe our results reflect the true trend in DBT effective focal spot size increase between models and tube motion techniques, the actual numbers may vary slightly if measured on different units of the same manufacturer and model. Though we couldn't quantify the variability between multiple units of the same model/manufacturer, the primary factors that impact focal spot blur, such as tube motion and geometry, are consistent between units. Further studies may quantify the variability between units, though we expect this to be relatively small compared to the variability between models.

## 5 CONCLUSION

DBT acquisitions involve trade‐offs between angular range, blur, and imaging time, with manufacturers adopting different optimization strategies. While step‐and‐shoot reduced blur but increased imaging time, and continuous motion increased blur and reduced imaging time, we found that the flying focal spot technique reduced both imaging time and blur.

## AUTHOR CONTRIBUTIONS

Colin Schaeffer: Concept and design, measurements, data analysis, manuscript preparation. Katie Hulme: Measurements, manuscript preparation. Ashley Rubinstein: Concept and design, measurements, manuscript preparation, research management and supervision.

## CONFLICT OF INTEREST STATEMENT

The authors have no relevant conflicts of interest to disclose.

## Data Availability

Data is available upon request.
